# Activation of NK cells and disruption of PD-L1/PD-1 axis: two different ways for lenalidomide to block myeloma progression

**DOI:** 10.18632/oncotarget.15234

**Published:** 2017-02-09

**Authors:** Massimo Giuliani, Bassam Janji, Guy Berchem

**Affiliations:** ^1^ Laboratory of Experimental Cancer Research, Department of Oncology, Luxembourg Institute of Health, Luxembourg City, Luxembourg; ^2^ Department of Hemato-Oncology, Centre Hospitalier du Luxembourg, Luxembourg City, Luxembourg

**Keywords:** lenalidomide, multiple myeloma, NK cells, PD-L1/PD-1, immunotherapy

## Abstract

Natural Killer (NK) cells play a critical role against tumor cells in hematological malignancies. Their activating receptors are essential in tumor cell killing. In Multiple Myeloma (MM) patients, NK cell differentiation, activation and cytotoxic potential are strongly impaired leading to MM escape from immune surveillance in tissues and bone marrow. Mechanisms used by MM to affect NK cell functions are mediated by the release of soluble factors, the expression of activating and inhibitory NK cell ligands, and the expression of immune check-point inhibitors. Lenalidomide represents an efficient clinical approach in MM treatment to improve patients’ survival. Lenalidomide does not only promotes tumor apoptosis, but also stimulates T and NK cells, thereby facilitating NK-mediated tumor recognition and killing. This occurs since Lenalidomide acts on several critical points: stimulates T cell proliferation and cytokine secretion; decreases the expression of the immune check-point inhibitor Programmed Death-1 (PD-1) on both T and NK cells in MM patients; decreases the expression of both PD-1 and PD-L1 on MM cells; promotes MM cell death and abrogates MM/stromal microenvironment cross-talk, a process known to promote the MM cell survival and proliferation. This leads to the inhibition of the negative signal induced by PD-1/PD-L1 axis on NK cells, restoring NK cell cytotoxic functions. Given the importance of an effective immune response to counteract the MM progression and the promising approaches using anti-PD-1/PD-L1 strategies, we will discuss in this review how Lenalidomide could represent an adequate approach to re-establish the recognition against MM by exhausted NK cell.

## NK CELL-MEDIATED TUMOR SURVEILLANCE AND MULTIPLE MYELOMA

NK cells play a critical role in cancer surveillance, and their cytotoxic functions are regulated by a balance between the expression of activating and inhibitory receptors [1–4]. Activating receptors includes Natural Cytotoxic Receptors (NCRs), such as NKp30, NKp44 and NKp46, NKG2D, DNAX Accessory Molecule-1 (DNAM-1) and several co-stimulatory receptors such as LFA-1, NKp80 and CD244 (2B4). The main group of inhibitory receptors are the Killer Inhibitory Receptors (KIRs) and NKG2A, specific for Major Histocompatibility Complex (MHC) class I molecules. Activating receptors initiate granule-dependent killing where Perforin and Granzymes are released by NK cells followed by synapse formation with the target. NK cells play also a critical role in autologous stem cell transplantation in several hematological malignancies, as described in the section [5–10]. Advanced findings have demonstrated that the number of NK cells isolated from patients with hematological malignancies including Multiple Myeloma (MM) is strongly decreased and their cytotoxic functions are seriously impaired [11–18]. An overview of pathogenesis and therapeutic strategies in MM is reviewed in the following references [19–22]. Importantly, NK cells isolated from MM patients display a significant down-regulation of CD16, DNAM-1 and 2B4/CD244 expression, associated with impaired killing abilities [14–18, 23, 24]. Recently it has been shown that NK cells isolated from MM patients display decreased expression of the activating receptor CD161 and an increased expression of KIRs, which contribute to the impairment of NK cell cytotoxic functions [25, 26].

## MECHANISMS USED BY MM CELLS TO SUPPRESS NK CELL FUNCTIONS

### Immune check-point inhibitors: role of PD-L1/PD-1 axis

Hematological malignancies develop several strategies to impair the immune response or to modulate the tumor microenvironment. Acute Myeloid Leukemic (AML) cells express several co-stimulatory molecules such as CD80 and CD86 to interact with CTLA4 (cytotoxic T-cell antigen-4)-expressing T cells [27]. In addition, AML cells express immune inhibitory check-points and HLA class I molecules, which contribute to the anti-tumor response exhaustion and immune escape [27, 28]. Different approaches have been proposed to harness the anti-tumor response against AML cells, such as antigen-targeted therapies, inhibitory check-points modification and cytokine therapies [27]. Similarly, it has been shown that malignant cells from Chronic Lymphocytic Leukemia (CLL) patients impair NK cell cytotoxic functions, thus facilitating NK cell anergy and tumor progression [29]. In addition, compared to healthy donors, CLL cells secrete elevated amount of soluble NK activating ligands, which contribute to the impairment of NK killing activity [30]. Furthermore, it has been also shown that stromal cells, nurse-like cells (NLC) and follicular dendritic cells (FDC) in the tumor microenvironment play a major role in CLL progression and drug resistance [29, 31]. In the last decade, several immunotherapies have been validated in CLL patients, including Immunomodulatory drugs, monoclonal antibodies, Bi-Specific T cell Engagers (BiTE^®^), immune check-point inhibitors and chimeric antigen receptor (CAR) T cells [32].

**Figure 1:**
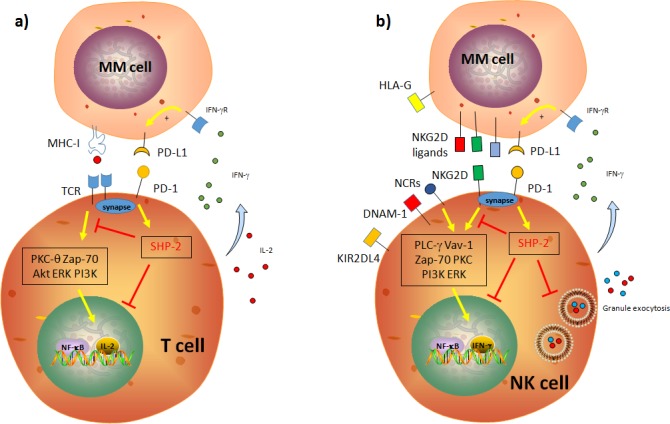
Schematic representation of the impact of the PD-1/PD-L1 axis on T and NK cell cytotoxic functions **a.** PD-1 blocks the activating cascade induced by the T-cell receptor (TCR) and the activating receptors in T cells and NK cells, respectively. PD-1 activates Src-homology 2-containing tyrosine phosphatase (SHP-2), which interfere with PKC-θ, PI3K, ERK and AKT activation and signaling, critical for T cell proliferation. **b.** Similarly, PD-1-mediated SHP-2 activation inhibits PKC-θ, PI3K and ERK activation, critical for NK cell functions. This leads to an impaired proliferation, cytotoxicity, granule exocytosis and IFN-γ secretion in NK cells.

Dendritic cells (DC) in MM patients display decreased co-stimulatory molecules expression and functions, and therefore are in an immature state. These immature DC are responsible for affected T cell activation and migration [26, 33, 34]. In addition, Sponaas et al., reported that DC and plasma cells in myeloma patients express PD-L1 (Programmed death ligand 1), which is correlated with an impairment of anti-tumor response [35]. Interestingly, it has been reported that DC play a dual opposite role as promoter of CD8+ T cells-mediated MM cell killing and as facilitator of MM cell resistance to CD8(+) T-cell killing [36]. Additionally, a positive effect in DC activation mediated by Lenalidomide treatment has been observed in both MM patients [37] and murine MM model [38]. Macrophages represent an important population in the MM microenvironment [39]. MM cells secrete inflammatory factors to recruit macrophages in the tumor site; in turn, macrophages are able to differentiate from M1 (classically activated, pro-inflammatory) to M2 (alternatively activated) phenotype, secreting factors to promote survival and drug resistance in MM cells [39, 40]. Interestingly, the stromal microenvironment promote this differentiation by secreting soluble factors such as CCL2 and IL-6 [41, 42]. Concerning the T cell subsets, change in CD8/CD4 ratio in T cells has been observed in MM patients, where most of T cells display reduced expression of co-stimulatory molecules, such as CD28, and increased expansion of Treg and Th17 subset, which in turn promotes MM cell growth [33, 34]. Among these strategies, a critical role in MM progression is played by the secretion of soluble factors as Prostaglandin-2 (PGE-2) and Transforming Growth Factor (TGF-β), or by the expression of immune check-point inhibitors [18, 33, 43–45]. Advanced findings have shown in fact that the immune check-point inhibitor PD-L1 play a major role in MM escape from immune cells [18, 33, 43, 46]. PD-L1 can be expressed by solid tumors and hematological malignancies to inhibit T and NK cell functions and, paradoxically, its expression can be increased by the IFN-γ secreted by immune cells, persisting its inhibitory effect [47–49]. In contrast to healthy plasma cells, those derived from MM patients express PD-L1 and therefore participate to the inhibition of Cytotoxic T Cells (CTL) proliferation and cytolytic functions [46, 50–56]. Notably, Wang et al., recently reported that PD-L1 can be also found as soluble form in MM patients, which could represent a potential marker for diagnosis and therapy [57]. The PD-L1 receptor PD-1 is expressed on activated T and NK cells [58]. Triggering PD-1 blocks the activating cascade induced by the T-cell receptor (TCR) and the activating receptors on T cells and NK cells, respectively [59, 60]. PD-1 activates Src-homology 2-containing tyrosine phosphatase (SHP-2), which interfere with PKC-θ, PI3K, ERK and AKT activation, critical for T cell proliferation (for a detailed review see [59, 60]) (Figure 1a). Persistent expression of PD-1 in T cells leads not only to T cell exhaustion and an impaired T cell-mediated immunosurveillance, but also to the development of regulatory T cells (Treg) population [48, 61]. Similarly, PD-1-mediated SHP-2 activation inhibits activating receptors-induced cytotoxicity, granule exocytosis and IFN-γ secretion in NK cells [4, 48, 61] (Figure 1b). Recently, it has been reported that PD-1 microclusters can be found in the NK immunological synapse (NKIS), thus impairing NK cell recognition of target cells [62]. Interestingly, it has also been shown that cytokines such as IL-18 could up-regulate PD-1 expression on NK cells in the lymph nodes, thus promoting metastases dissemination of NK cell-dependent tumors [63]. It is noteworthy that PD-1 is absent on NK cells isolated from healthy donor but it is expressed on those from MM patients [50]. Furthermore, NK and T cells stimulated with an anti-PD-1 antibody CT-011 restored the cytolytic ability of NK to kill target cells [46, 50, 54, 55]. Additionally, clonal T cells in MM can be hypo-responsive and fail to respond to several cytokines [64, 65]. Interestingly, these T-cell clones isolated from MM patients have normal TCR signaling but a defective p-SMAD pathway, which suppress cell proliferation. In particular, these T-cell clones displayed low levels of PD-1, which could explain their telomere-independent senescence [64, 65]. These results point out to the fact that PD-1^low^ T-cell clones fail to be fully activated by an anti-PD-1 therapy in MM, leading to a partial response in MM patients [65, 66].

Advanced evidences demonstrate that BM stromal cells isolated from MM patients display several abnormalities, compared to healthy donors [67–69]. The consequence is an uncontrolled MM progression, survival, growth and drug resistance, associated with angiogenesis and tumor escape [19, 70–72]. In turn, MM cells induce stromal cells to promote a pro-angiogenic and an immuno-suppressive milieu. Interestingly, Ponzetta et al., have recently demonstrated that MM cells and tumor microenvironment influence NK cell trafficking in the bone marrow (BM), impairing NK cell-mediated tumor recognition [73]. Mechanisms involved in this evasion are the down-regulation of CXCR3 and CXCR4 on NK cells and the up-regulation of chemokines secretion by the tumor microenvironment such as CXCL9 and CXCL10. Interestingly, MM cells cultured with stromal cells express high levels of PD-L1 [71, 74]. The consequence is that PD-L1-expressing MM cells interact with the PD-1 expressed by immune cells, thus affecting T and NK cell activation and supporting MM progression and immune-escape [50]. These results suggest that the PD-1/PD-L1 axis expressed on MM cells, stromal and immune cells, play a critical role not only in supporting MM progression and survival, but also in protecting cancer cells from effector cells [50].

### Activating NK ligands

Another major mechanism used by MM cells to escape to NK cell-mediated attack is the modulation of the expression of NKG2D [MHC class I related chain A/B (MICA/B), UL16-binding proteins (ULBPs)] and DNAM-1 (PVR and Nectin-2) ligands on their surface [3, 18, 75]. Notably, the down-regulation or the release of MICA expression facilitate tumor cell escape [13, 17, 23, 76–81]. Furthermore, Jinushi and colleagues have shown that plasma cells from MM patients express low level of MICA at their surface and significant high level of soluble MICA (sMICA), whereas plasma cells from MGUS patients have opposite expression of MICA [82]. Consequently, sMICA could represent a prognostic factor in MM patients [81]. In addition, NK cells derived from MM patients displayed a reduced expression of NKG2D, but a large amount of sMICA in the patients’ plasma. Interestingly sMICA was not correlated with the reduced expression of NKG2D on NK cells, suggesting that MM escape is promoted by a direct interaction between NK and MM cells, rather than by sMICA secreted by MM cells themselves [83].

Furthermore, the up-regulated expression of MHC class I molecules and the decreased expression of Fas have been described to protect MM cells from NK cell lysis [14, 16]. Importantly, in early stages of the MM pathogenesis, the escape from NK cells seems to be associated with a down-regulation of the activating ligands rather than an increase of inhibitory ligands. However, in late-stage, MM are rather protected from NK cell lysis by a high expression of HLA class I molecules [13, 14, 16, 26, 82].

## LENALIDOMIDE AS PROMISING STRATEGY TO RESTORE IMMUNE RESPONSE AND IMPROVE PATIENTS SURVIVAL

### Lenalidomide and MM cells survival

MM is a hematologic cancer characterized by an accumulation of terminally differentiated plasma cells in the BM [21, 84]. Despite the use of several therapeutic strategies MM is still incurable disease and numerous patients relapse and/or develop resistance to current therapies. The choice of drug treatment depends on the tumor progression and the age of the patient. Strategies include, among others, (a) Bortezomib/Thalidomide/Dexamethasone (VTD) in relapsed refractory myeloma, (b) Vincristine/Adriamycin/Dexamethasone (VAD) in patients which will receive Stem Cell Transplantation (SCT), (c) Melphalan/Prednisone combination in patients who are ineligible for an Autologous SCT, (d) Melphalan/Prednisone/Thalidomide or (e) Bortezomib/Melphalan/Prednisone [21, 84–88]. Interestingly, several groups reported that the number, the activation and the cytotoxic functions of NK cells are increased during and after SCT. This is associated with an overall survival in MM patients [5], even in T cell-depleted allografts [6, 89]. In addition, following autologous SCT, NK cells display higher expression of CD57 and KIRs, compared to the same cell analyzed before or at later time points after SCT. Although these NK cells also strongly express KIR2DL2/3/S2 and KIR3DL1, associated with a more immatures characteristics, they show granule exocytosis and secretion abilities [90]. In addition, Htut and colleagues showed that NK cells in MM patients undergoing hematopoietic cell transplantation (HCT) displayed a decreased expression of the TNF receptor OX40 (CD134) [91]. Interestingly, Lenalidomide treatment during and after SCT improved anti-myeloma activity (NCT00778752) [92, 93] by increasing the proliferation and NKp44 expression on NK cells [91, 92], which are associated with a significant down-regulation of CTLA-4 expression [91].

**Figure 2:**
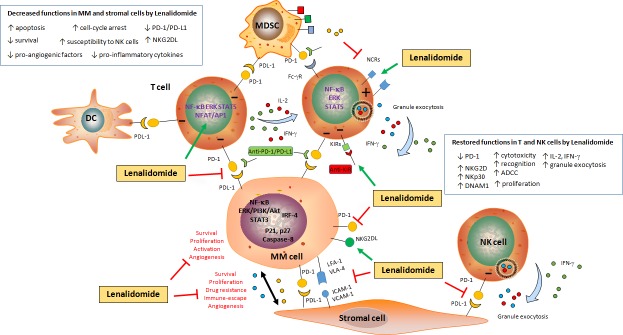
Schematic representation of the impact of Lenalidomide on MM cell survival and immune escape Lenalidomide induces apoptosis (by increasing p21, p27 and Caspases expression) and impairs survival (by blocking several pathways such as NF-κB and PI3K/Akt and inducing cell-cycle arrest) in malignant plasma cells. Additionally, Lenalidomide disrupts the MM/BMSC cell cross-talk, by inhibiting TNF-α-induced adhesion molecules (VLA-4, LFA-1, ICAM-1 and VCAM-1) expression on both MM and stromal cells, as well as cytokine secretion (i.e. IL-6, TGF-β and IGF-1) and VEGF-mediated angiogenesis. Lenalidomide down-regulates the expression of PD-1 on MM cells and the expression of PD-L1 on both stromal and MM cells, thus inhibiting the vicious circle involved in the impairment of the immune response. Lenalidomide also activates T cells to secrete IL-2 and IFN-γ, and down-regulates the expression of PD-1 on T and NK cells. This restores NK cell activation, as shown by the increased granule exocytosis (Perforin and Granzyme B) and ADCC, re-establishing cytotoxic functions against tumor cells. In addition, Lenalidomide can be used associated with CT-011 (an anti-PD-1 antibody) to restore immune cell functions.

In the last decade new approaches such as IPH-2102 (anti-KIR) mAb therapy and immune check-points inhibitors (ipilimumab (anti-CTLA-4) [48, 49, 94, 95] have made further progresses in solid tumors and hematological malignancies. However, their impact on MM cell functions and the NK/MM interaction has not been completely investigated. A similar argument could be used for other drugs such as the proteasome inhibitors Bortezomib and Carfilzomib (PR-171) and Histone Deacetylases inhibitors (HDACi), which have been demonstrated to improve anti-tumor response [12, 96, 97]. The survival rates of relapsed MM patients has been significantly improved since the introduction of Immunomodulatory drugs (IMiDs) including Thalidomide, Lenalidomide and more recently Pomalidomide [86, 97]. Thalidomide was described as anti-angiogenic, anti-tumor and immune modulatory agent affecting many cell types [97–100]. These properties have contributed to re-approved Thalidomide by the Food and Drug Administration (FDA) for MM treatment. Similarly, Pomalidomide (Pomalyst™) has been approved by FDA in 2013 due to its anti-tumor properties characterized by increasing NK cell activation, down-regulating osteoclastogenesis and inhibiting the interaction between stromal and myeloma cells [96, 97, 101]. The introduction of Lenalidomide (CC-5013, Revlimid^®^) as new clinical approach improved the median survival of MM patients, even in those developing resistance and disease relapse [102–105]. Following its approval in 2006 by FDA in MM treatment, a plethora of clinical trials registered in www.clinicaltrials.gov demonstrated a strong anti-tumor effect of Lenalidomide when administrated alone or combined with others drugs [22, 102, 103, 106–108]. Notably, Lenalidomide displays anti-tumor abilities on malignant plasma cells, affecting multiple mechanisms involved in tumor development and survival [97]. For example, Lenalidomide display anti-osteoclastogenic properties and induces cell cycle arrest and increases the expression of Cyclin-dependent kinase (CDK) inhibitors on MM cells, thus inhibiting their proliferation and promoting their apoptosis [97, 109–111] (Figure 2). Recent findings also show that Lenalidomide partially reversed the exhaustion of effector cells promoted by stromal microenvironment Myeloid-derived suppressor cells (MDSC) [112] and DC [35] (Figure 2). Lenalidomide also acts on tumor microenvironment, by disrupting the MM/stromal cells cross-talk. This leads to a decreased secretion of pro-angiogenic and anti-inflammatory molecules, and down-regulated expression of both PD-1 and PD-L1 expression in MM cells, constitutively expressed or induced by the stromal microenvironment [50, 54, 74]. These results highlight the critical role of BM microenvironment on MM progression, and the importance to develop anti-tumor approaches based on the PD-1/PD-L1 complex [50, 71, 74]. The positive impact of PD-1/PD-L1 axis in MM eradication has been also confirmed *in vivo* in a myeloma murine model (5T33) [54, 113]. Authors demonstrated that PD-1/PD-L1 blockade with a PD-L1-specific Ab elicits rejection of a murine myeloma when combined with lymphodepleting irradiation [113]. In addition, T cells from myeloma-bearing mice up-regulate their PD-1 expression in response to multiple myeloma [54]. Interestingly, these PD-1-expressing CD8^+^ T cells, although activated, do not secrete inflammatory cytokines and they undergo to apoptosis. It has been reported that these lymphocyte express TIM-3 (T-cell immunoglobulin and mucin-domain containing-3), a marker synonimous of cell exhaustion [114, 115]. Of note, the blockade of PD-L1 during vaccine administration resulted in improved vaccine efficacy. Together, these results are very interesting since, as discussed above, Lesokhin et al., shown that T-cell clones PD-1^low^ lead to a partial response in MM patients with an anti-PD-1 therapy [66]. The positive effect of Lenalidomide on MM killing has also been recently reported by Ray and colleagues. They demonstrated that IMiDs combined with ACY-1215 (Ricolinostat), Bortezomib, anti-PD-L1 antibody or Toll-like receptor agonists strongly increased the anti-tumor response [116]. In this case, Lenalidomide enhanced the effect of PD-1/PD-L1 blocking on NK cell-mediated tumor killing. Interestingly, the positive combination of Pembrolizumab/Dexamethasone with Lenalidomide [117] and Pomalidomide has been also reported in MM patients [118] (NCT02289222). A summary of ongoing and completed Clinical Trials in hematological malignancies including MM using PD-1 [Pidilizumab (CT-011) or Pembrolizumab] and PD-L1 (Atezolizumab) can be found in www.clinicaltrials.gov and [47–49, 119]. The Table 1 summarizes current recruiting Clinical trials using Lenalidomide combined with anti-PD-1/PD-L1 antibodies in hematological malignancies treatment.

**Table 1:** MM, Multiple Myeloma; MDS,Myelodysplastic Syndrome; NHL, Non-Hodgkin's Lymphoma; FL, Follicular Lymphoma; PD-L1, Programmed Death Ligand-1

Study	Therapy	Disease	Clinical trial	Status
A Study of Atezolizumab (Anti-Programmed Death Ligand 1 [PD-L1] Antibody) Administered With or Without Lenalidomide in Participants With Multiple Myeloma (MM)	Lenalidomide Atetolizumab	MM	NCT02431208	recruiting
A Study of Pembrolizumab (MK-3475) in Combination With Standard of Care Treatments in Participants With Multiple Myeloma (MK-3475-023/KEYNOTE-023)	Lenalidomide Pembrolizumab Dexamethasone	MM	NCT02036502	recruiting
Study of Lenalidomide and Dexamethasone With or Without Pembrolizumab (MK-3475) in Participants With Newly Diagnosed Treatment Naive Multiple Myeloma (MK-3475-185/KEYNOTE-185)	Lenalidomide Dexamethasone Pembrolizumab	MM	NCT02579863	recruiting
A Trial of Pembrolizumab (MK-3475) in Participants With Blood Cancers (MK-3475-013)(KEYNOTE-013)	Pembrolizumab Lenalidomide	MM NHL Lymphoma MDS	NCT01953692	recruiting
Phase 2 Multi-center Study of Anti-PD-1 During Lymphopenic State After HDT/ASCT for Multiple Myeloma	Lenalidomide Pembrolizumab	MM	NCT02331368	recruiting

As described above, MM cells express activating NK cell ligands involved in the recognition of NK cells but not in the killing [12, 77–79, 82]. Fionda et al., recently show that Lenalidomide increases the expression of NKG2D and DNAM-1 ligands on both malignant plasma cells and MM cell lines leading to NK cell interaction and tumor cell killing [120]. Interestingly, authors also demonstrated that the negative modulation of Cerebron, Ikaros (IKZF1), Aiolos (IKZF3) and Interferon-Regulatory Factor (IRF)-4 induced by Lenalidomide was critical to promote the NKG2D ligands expression on MM cells (Figure 2). It is worthy to note that the second-generation of Proteasome Inhibitor Carfilzomib enhanced the sensitivity of MM cells to NK cell-mediated lysis [121]. In addition, Carfilzomib-activated NK cells also displayed an increased cytotoxic granule secretion and cytotoxicity which was correlated to the decreased expression of HLA class I in Carfilzomib-treated MM cells. Unfortunately, the impact of Lenalidomide in HLA class I expression on MM cells and the consequence on anti-tumor response has not been investigated so far.

### Lenalidomide restores NK cells cytotoxicity

Lenalidomide displays immunomodulatory properties by inducing IL-2 and IFN-γ secretion by T cells, Antibody-dependent cell-mediated cytotoxicity (ADCC) as well as NK cell cytotoxic functions [12, 92, 97, 100, 109, 122–125] (Figure 2). In addition, Lenalidomide increases co-stimulatory receptors expression on NK cells, as CD16 and Lymphocytes Function-associated Antigen (LFA-1) [12, 97, 126–129]. Of note, a large heterogeneity exists in *in vitro* experimental protocols that depends on NK cell sources (total PBMC against purified NK cells), IL-2 and drug concentration, treatment period, targets. Notably, Lenalidomide down-regulates PD-1 expression on T cells isolated from MM patients, allowing the cytotoxic restoration of their cytotoxicity [127]. Intriguingly, Daguet et al., reported that Lenalidomide affects the secretion of IFN-γ by NK cells isolated from healthy donors, and decreases activating receptors expression on NK cells [130]. These findings could explain why Lenalidomide somehow does not directly supports NK cell activation. Interestingly, an opposite effect is observed in CLL patients, since Lenalidomide-stimulated NK cells display a reinforced cytotoxic activity and increased proliferation [125, 131] and a repaired immunological synapse, critical for NK cell-mediated tumor surveillance [132]. As already discussed, Benson et al., reported that IPH2101 (an anti-KIR) prevents negative signals by KIRs expressed on NK cells [133]. Importantly, IPH2101 can be combined with Lenalidomide which, by improving NK cell activation and increasing NK cell ligands on MM cells, contributes to enhance the *in vivo* anti-tumor response. Interestingly, the same group have recently published results about the effect of Lenalidomide combined with IPH2101 (without corticosteroids) in relapsed/refractory patients in a Phase I trial [134]. It is important to mention that, although improving MM patients’ survival [22, 107, 108, 135–137], Dexamethasone administration could be at detrimental of the immune surveillance against tumor cells [109, 123, 125, 138, 139]. Advanced findings have in fact demonstrated that Dexamethasone decreases the NKG2D, NKp30 and NKp46 expression on NK cells, as well as the secretion of IL-2 and IFN-γ by NK cells [109, 123, 125]. In addition, Dexamethasone decrease IL-2 and IFN-γ secretion in normal PBMCs, as well as activated NK cell-release of Granzyme B, by antagonizing the stimulatory capacity of Lenalidomide in both T and NK cells [109].

## CONCLUSIONS

The positive impact of Lenalidomide based therapies has been observed in several hematological malignancies. For example, NK cells stimulated with Lenalidomide display a reinforced cytotoxic potential in CLL patients [125, 131, 132, 140]. In AML patients, NK cells show a reduced target killing since their ability to form an efficient immunological synapse is impaired [141]. Interestingly, lytic granules polarization in the immunological synapse was significantly restored after Lenalidomide treatment. A positive action of Lenalidomide in restoring synapse formation, ADCC, and cytotoxic functions in NK cells have been also reported in B-cell Non Hodgkin Lymphoma patients [142, 143].

By promoting cytokine secretion and activating receptors stimulation on NK cells, associated with an inhibition of the PD-1/PD-L1 axis to disrupt the MM/stromal cell cross-talk and the immune response exhaustion, Lenalidomide restores NK cell functions in MM patients. Furthermore, Lenalidomide can be combined with monoclonal antibodies (mAbs) such as CT-011 (anti-PD-1) [50] to enhance their own positive effect on NK cells. However, certain drugs used in MM therapy such as Dexamethasone interfere with Lenalidomide-induced NK cell activation *in vitro*. Thus, a more precise understanding of the molecular mechanisms induced by a drug on the immune system should be verified before applying it in MM patients. In conclusion, given the importance of NK cells in cancer surveillance and in autologous SCT in MM patients, Lenalidomide is currently the more complete treatment (alone or combined with anti-PD-1/PD-L1 antibodies or other drugs) able to restore exhausted NK cell cytotoxic functions and to impair MM cell survival and immune-escape. These findings support the fact that Lenalidomide represents an adequate strategy in MM patients to reinforce immune anti-tumor activity.
